# Fournier's Gangrene in Children: Report on 7 Cases and Review of Literature

**Published:** 2014-07-20

**Authors:** Mohsen Rouzrokh, Atossa Tavassoli, Alireza Mirshemirani

**Affiliations:** 1 Pediatric Surgery Research Center, Shahid Beheshti University; 2 Pediatric Neurologist, Ali-Asghar Children's Hospital, Iran University of Medical Sciences, Tehran, Iran

**Keywords:** Gas gangrene, Perianal abscess, Fournier's gangrene, Necrotizing fasciitis

Fournier's gangrene (FG) is named after Alfred Fournier who described it as a rapidly progressive necrotizing fasciitis of the perineum and external genital organs in young adults with unknown cause in 1883^[^^1^^]^. The disease mostly affects men between 50 and 60 years of age and is considered uncommon in the pediatric age group^[^^2^^,^^3^^]^. Here we present seven cases of FG who referred to our hospital in. 

 From 2009 to 2013, seven cases with FG were treated in our center. They have been treated with urgent aggressive surgical debridement and intensive care support, although antibiotic therapy, correct postoperative wound management and complete excision of all necrotic tissue was performed successfully, three patients died during treatment. Colostomy was performed in 5 patients, and one case underwent urinary diversion.

 In average 2 (1 to 5) debridement steps were undertaken. Colostomy was performed in 5 patients. Urinary diversion was performed in one patient. Wound dressing with 0.5% H_2_O_2_ and 1% citric acid solution under general anesthesia were performed daily. Culture of the debris tissues revealed the offending organisms to be Kellebsila, Streptococci, E-coli and Pseudomonas. Five cases survived after primary and subsequent reconstructive treatment; three cases died despite appropriate treatment due to sepsis and multi organ failures. A summary of findings in 7 cases is provided in Table1.

 Controversies in defining FG have emerged. Some suggested that the name Fournier's gangrene should be reserved for those cases in which a source of bacteria was not demonstrable^[^^4^^]^. Despite this, most authors still use the term broadly for necrotizing fasciitis of the perieneal region. The predisposing factors for FG include abscesses, omphalitis, and diaper rash, surgical procedures like circumcision and herniorhaphy, burns, insect bites, anorectal trauma, and nephritic syndrome^[^^5^^,^^6^^]^. Others include systemic disorders like immunocompromised states, or hematologic malignancies^[^^6^^]^. In the second case presented both omphalitis and an undiagnosed immunodeficiency syndrome could be named as predisposing factors. The underlying cause of Fournier's gangrene may lie in urinary tract, colorectal or local skin and the usual offending organisms are E Coli, Bacteroids, Staphylococci, Streptococci, and Clostridia, etc. Infection is frequently polymicrobial gram negative organisms, gram positive organisms and even anaerobes^[^^7^^]^. In our patients Pseudomonas proved to be the causative organism. This was not surprising, as it is not one of the top causative agents. 

 The management of FG includes aggressive resuscitation with I.V. fluid, blood and broad spectrum parenteral antibiotics. Surgical debridement of necrotic tissues will control spread of infection and induce reduction of systemic toxicity. When the source of infection is from the ano-rectal region or when urinary extravasation or peri-urethral inflammation is present, urinary or fecal diversion is indicated to reduce contamination and allow wound healing to take place^[^^8^^]^. Two of our patients underwent extensive debridement and while urinary and fecal diversion was performed in one of the patients, the other one only received fecal diversion, both patients received proper antibiotic treatment.

 The diagnosis and treatment in the FG patient does not always terminate to death in infants, and several cases of Fournier's gangrene in infants have been treated successfully by surgical debridement and parenteral antibiotics which have been reported^[^^7^^]^. Some have even suggested that the prognosis of FG is more favorable in children than in adults^[^^3^^]^. Despite aggressive treatment three of our patients did not survive. This might be due to miss-management of these cases.

 Although FG is not common in children and it may be fetal but early diagnosis is very important. Antibiotic therapy, early wide surgical debridement and early fecal diversion are the most part of treatment to preserve life.

**Table 1:** Data at onset and prognosis in 7 patients with FG

**Parameter**	**Case 1**	**Case 2**	**Case 3**	**Case 4**	**Case 5**	**Case 6**	**Case 7**
**Age**	6 m	5 m	8 y	6 m	6 y.	5 m	8 y.
**Sex**	Male	Male	Male	Female	Male	Female	male
**Underlying disease**	Buttock abscess	Perineal abscess	Car accident & perineal rupture	LADS & genital abscess	AML & gluteal cellulitis	ALL & anal fissure	Perineal Cellulitis & Fecal Fistula
**WBC **(%PMN)	12000 (90%)	18000 (85%)	15000 (95%)	22000 (90%)	2500(67%)	1250 (70%)	17000 (87%)
**Hemoglobin**	9.1	7.8	6	6	9	8.2	8.1
**Platelet count**	45000	22000	161000	75000	86000	70000	245000
**BUN**	23	36	8	12	16	21	40
**Creatinine**	0.6	1.2	0.5	0.8	1.4	1.4	1.3
**Sodium (Na)**	125	128	136	130	135	132	128
**Potassium (K)**	2.9	4	3.4	3.6	4.2	3.8	4.2
**PT (sec)**	45	13	14	14	22	33	13
**PTT (sec)**	120	30	34	32	45	86	32
**CRP**	4+	2+	2+	4+	2+	3+	3+
**ESR**	1	40	102	120	68	54	87
**Blood culture**	Pseud. Aeru.	E. coli	Contaminated	E. coli	Strep	Strep	Negative
**Wound culture**	Pseud. Aeru.	E. coli Pseud. Aeru.	Pseud. Aeru.	Mixed	Pseud. Aeru.	Pseud. Aeru. Candida Alb.	Mixed
**Urine culture**	Negative	Negative	Negative	E. coli	Negative	Negative	E. coli
**Prognosis**	Alive	Death	Alive	Death	Alive	Death	Alive

WBC: white blood cell; BUN: Blood urea nitrogen; PT: Prothrombin time; PTT: Partial thromboplastin time; CRP: C-reactice protein; ESR: erythrocyte sedimentation rate; AML: Acute myeloid leukemia; ALL: Acute lymphoblastic leukemia; LADS: Leukocyte adhesion deficiency; Pseud. Aeru.: Pseudomonas Aeruginosa

This study was financially supported by the office of the Vice Chancellor for Clinical Research Development Center of Mofid Children's Hospital.
